# Genome sequence analysis reveals potential for virulence genes and multi-drug resistance in an *Enterococcus faecalis 2A* (XJ05) strain that causes lamb encephalitis

**DOI:** 10.1186/s12917-019-1936-3

**Published:** 2019-07-08

**Authors:** Yingjin Chai, Xiaoxiao Gu, Qin Wu, Bingjiao Guo, Yayin Qi, Xiaolan Wang, Xia Zhou, Jie Li, Mengli Han, Fagang Zhong

**Affiliations:** 1 College of Animal Science and Technology, Shihezi University, Shihezi, 832000 Xinjiang China; 2 State Key Laboratory for Sheep Genetic Improvement and Healthy Production, Xinjiang Academy of Agricultural and Reclamation Science, Shihezi, 832000 Xinjiang China

**Keywords:** Lamb encephalitis, *E. faecalis*, Whole genome sequencing

## Abstract

**Background:**

Enterococcus is an important component of normal flora in human and animals, but in recent years, the pathogenicity of Enterococcus has been confirmed in clinical medicine. More and more animal infections have been reported in veterinary clinics. For the last decades, outbreaks of encephalitis in lambs have become much more common in Northern Xinjiang, China. Consequent studies have confirmed that these affected lambs had been commonly infected with *E. faecalis.* More than 60 *E. faecalis* were isolated from the brain of infected lambs, A highly virulent strain entitled *E. faecalis* 2A (XJ05) were selected, sequenced and analyzed.

**Result:**

Using whole genome sequence and de novo assembly, 18 contigs with NGS and annotation were obtained. It is confirmed that the genome has a size of 2.9 Mb containing 2783 protein-coding genes, as well as 54 tRNA genes and 4 rRNA genes. Some key features of this strain were identified, which included 7 predicted antibiotic resistance genes and 18 candidate virulence factor genes.

**Conclusion:**

The *E. faecalis* 2A (XJ05) genome is conspicuous smaller than *E.faecalis* V583, but not significantly different from other non-pathogenic *E. faecalis*. It carried 7 resistance genes including 4 kind of antibiotics which were consistent with the results of extensive drug resistance phenotypic, including aminoglycoside, macrolide, phenicol, and tetracycline. 2A (XJ05) also carried 18 new virulence factor genes related to virulence, hemolysin genes (*cylA, cylB, cylM*, *cylL*) may play an important role in lamb encephalitis by *E. faecalis* 2A (XJ05).

## Background

### Disease epidemiology

Enterococcus is naturally parasitic in human and animal intestines and it is often studied as a beneficial bacterium. At present, nosocomial infection of Enterococcus is becoming more and more serious in clinic due to various factors such as irrational use of antibiotics and immune preparations, which include urinary tract infection, sepsis, endocarditis, meningitis and pneumonia, etc. [1, 2].

There are also sporadic reports of other animal infections in veterinary clinics in recent years [3]. Xinjiang in Northwest China is a major sheep-raising province with the number of sheep ranking second in China. In the past few years, lambs from some large-scale sheep farms in Northern Xinjiang have suffered from an epidemic disease with a short course, no variety difference, mainly infecting lambs aged 20–40 days, causing cerebritis symptoms, sepsis and mortality of lambs up to 20–30%. More than 60 strains(11 strains were isolated during early outbreaks, and more than 50 strains were isolated from sporadic infections later) of bacteria were isolated from the brain of sick lambs collected without contamination, which were identified as *Enterococcus faecalis (E.faecalis)* in Enterococcus by VITEK-AMS32 and specific PCR in the author’s laboratory [4]. The clinical symptoms and pathological changes from the lambs which were artificially infected with the isolated bacteria were similar to those naturally infected lambs [5], bacterial antigens were localized in many tissues and organs including the brain of experimental animals (unpublished). It indicated that *E.faecalis* was the pathogen of lamb encephalitis prevalent in some large-scale sheep farms in Xinjiang.

### Organism

Enterococcus was initially isolated and identified from excrement using sodium azide, and this organism was found to be a non spore-forming Gram-positive coccus without flagella. This class of cocci was not only identified as one of the major species in the intestines of humans and animals, but is also widely distributed in mammalian gastrointestinal, respiratory, and reproductive tracts, even in soil and water. Some species of the genus have been used in the food industry.

Vancomycin-resistant Enterococci (VRE) has gradually become one of the most important pathogens of nosocomial infections since it was first isolated and reported in Duluich Hospital in 1988. The American Hospital Infection Surveillance System (NISS) has announced that Enterococcus is the second largest pathogen causing nosocomial infection [6]. So far, there were also reports about animal infections. As a representative species of Enterococcus, *E. faecalis* has a special and complex resistance pattern, multiple pathogenic factors and have attracted concern [7–9].

In recent years, a lamb infectious diseases characterized by neurological symptoms was found in northern Xinjiang, China. In the early outbreak of infection, 11 strains of *E. faecalis* were isolated and identified [10], more than 50 strains of *E. faecalis* were isolated and identified from the dead lambs (unreported) in the author’s laboratory, no other bacteria and viruses were detected. Because *E. faecalis* 2A (XJ05) has a wide range of drug resistance phenotypes and low LD_50_, it was selected for genome sequencing and made comparisons with other five *E. faecalis* from Genbank. The five *E. faecalis* can be categorized into pathogens, symbiotic bacteria, and probiotics. For example, *E. faecalis V583* and *E. faecalis OG1RF* are pathogens, *E. faecalis 62* and *E. faecalis D32* are symbiotic bacteria, while *E. faecalis Symbiofor 1* has been shown to have probiotic qualities [11–15].

### Genomic research progress

In veterinary clinic, the infected lambs showed neurological symptoms, even septicemia and eventually died. Some *E. faecalis* were only isolated from the brains of dead lambs and no other related viruses were detected, characteristic pathological changes of encephalitis were observed. *E. faecalis 2A* (XJ05) with extensive drug resistance and a lower LD_50_ was selected from more than 60 isolates.

**Fig. 1:**
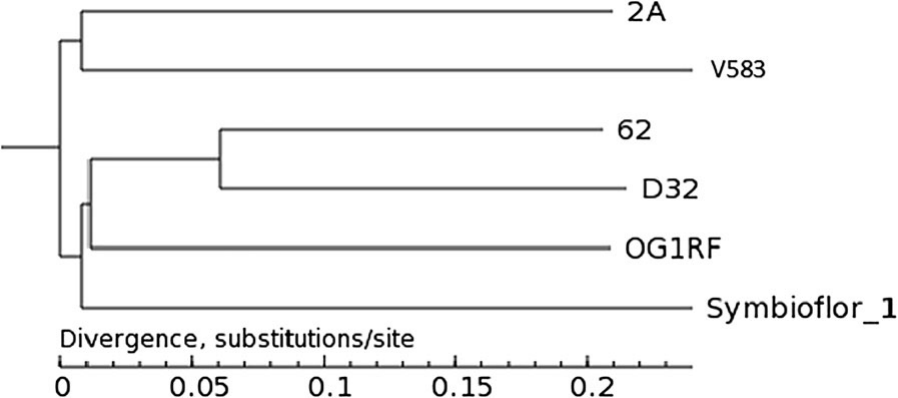
The evolutionary tree of six *E. faecalis* strains. *E. faecalis* 2A has high homology with *E. faecalis* V583, it has low homology with the other four *E. faecalis* strains

At present, there is a lack of systematic research about *E. faecalis* inducing lamb encephalitis. These *E. faecalis* strains were assessed using an evolutionary tree, according to mitochondria similarity. *E. faecalis 2A* (XJ05) and *E. faecalis V583* appeared to be relatively similar, with respect to another 4 compared strains (Fig. 1).

Five *E. faecalis* strains have provided poor genome sequence maps in succession, including *E. faecalis V583*, *E. faecalis D32*, *E. faecalis 62*, *E. faecalis OG1RF* and *E. faecalis Symbioflor 1*. However, *E. faecalis 2A* has no detailed genome information to date.

In this study, the assembled genome map was used to study common features and specific characteristics of the strain compared with other *E. faecalis* strains based on genome sequencing of *E. faecalis* 2A (XJ05). These results may provide some clues for lamb encephalitis prevention and treatment by revealing *E. faecalis* virulence-associated genes and resistance mechanisms.

## Results

### Genome assembly and annotation

2.93 million raw reads were gained with NGS technique with approximately 100 x coverage. After filtering low quality parts, removal of adapters, duplicated reads, and short inserts from the raw data, 2.89 Mb clean data were remained. Then, these sequences were assembled into 18 contigs using the SOAP de novo (version 2.04) with K setting at 83. The total sequence length reached 2.9 Mb, which was consistent with the previously estimated genome size. The GC content was 37.3%. By using Glimmer, 2787 CDS were predicted, which included 2729 protein-coding genes and 58 RNA genes (54 tRNA genes and 4 copies of 16S–23S-5S rRNA gene operons).

### Comparative genomics research

*E. faecalis 2A* (XJ05) genome was compared to *E. faecalis V583*, *E. faecalis D32*, *E. faecalis 62*, *E. faecalis OG1RF* and *E. faecalis Symbioflor 1*(Table 1). *E. faecalis 2A* (XJ05) was similar to the other 5 species of the same genus with GC content between 27.7–37.3%, and a CDS density between 0.941–0.969.

**Table 1:** Genome characteristic comparison of six *E. faecalis* strains

Strain	Genome length	G + C Content (%)	numbers of CDS	CDS density
*E.faecalis 2A* (XJ05)	2,915,744	37.3	2787	0.956 genes per kb
*E.faecalis V583*	3,218,031	37.5	3122	0.967 genes per kb
*E.faecalis D32*	2,987,450	37.4	2876	0.962 genes per kb
*E.faecalis 62*	2,988,673	37.4	2897	0.969 genes per kb
*E.faecalis OG1RF*	2,739,625	37.7	2579	0.941 genes per kb
*E.faecalis Symbioflor 1*	2,810,675	37.7	2686	0.956 genes per kb

*E. faecalis V583* was selected as a reference sequence and performed a whole-genome comparison among *2A*, *OG1RF*, *62*, *D32*, *symbioflor 1* and *V583* with BLAST 2.2.29. According to BRIG, they looked consistent with each other. However, there was also a lot of variation. At the position of approximately 0.5 Mb, *2A* (XJ05) and *V583* were quite different to *OG1RF*, *62*, *D32* and *symbioflor 1*. Furthermore, at the 2.25 Mb position, *V583* was unique compared to other members (Fig. 1).

From the inside to the outside, the former three circles were the location of the.

**Fig. 2:**
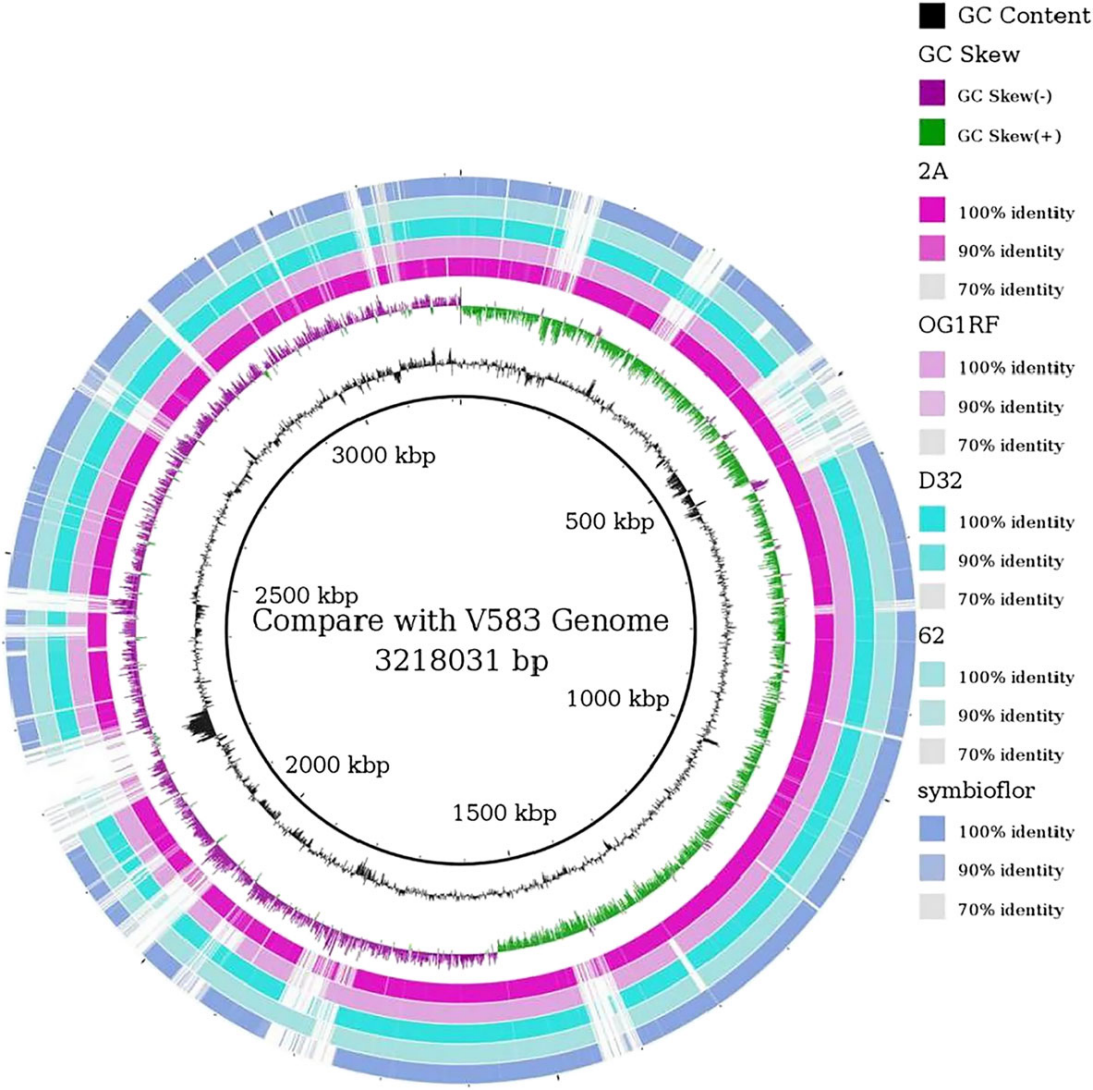
The results of comparing five bacteria and *V583.* The G + C and G + C skew similarity maps of other five bacteria were drawn based on V583. Circles 1–3 are *E. faecalis* V583, and 4–8 are *E. faecalis* 2A (XJ05), *E. faecalis* OG1RF, *E. faecalis D32*, *E. faecalis* 62, and *E. faecalis* symbioflor 1

*V 583* genome, GC content and GC skew respectively, while the latter five loops showed the match or mismatch sites (and degree) between *2A* (XJ05), *OG1RF*, *D32*,*62*, and *symbioflor 1* to *V583*. Color shades represented the range of similarity (100 > 90 and > 70%) (Fig. 2).

### Virulence genes and antibiotic resistance genes

In genome, 18 virulence factors of *E. faecalis 2A* (XJ05) were identified, without *fsrB*, *gelE* and *hylB*. Virulence factors *cylA* and *cylB* were specific to *2A*. In addition, there were 2 virulence factors, *cylM* and *cylL* co-owned by pathogenic *E. faecalis 2A* (XJ05) and *V583*. These 4 genes were located in the cytolysin operon. 13 virulence genes existed in all *E. faecalis* strains. They were *ace*, *efa*, *Afs*, *cad*, *cCF10*, *cOB1*, *hylA*, *ebpA*, *ebpB*, *ebpC*, *elrA*, *srtA*, *tpx* and *came*. Virulence factor *agg* was also observed in the presence of pathogenic bacteria *2A* (XJ05), *V583*, and probiotic bacteria *Symbioflor 1*. The gene *agg* was related to the aggregation substance (AS).

ResFinder 12 analysis (http://cge.cbs.dtu.dk/services/ResFinder/) showed that there were 7 antibiotic resistance genes in *E. faecalis 2A* (XJ05) (Table 2). They were related to resistance to four types of antibiotics, namely aminoglycoside, macrolide, phenicol, and tetracycline.

**Table 2:** Antibiotic resistance genes

Resistance gene	Identity	Query/HSP	Contig	Position in contig	Phenotype	Accession Number
*erm(B*)	100	738/738	Scaffold1	549,407..550144	Macrolide resistance	U18931
*aadE*	100	864/755	Scaffold1	554,596..555350	Aminoglycoside resistance	KF421157
*aac(6′)-aph(2″)*	100	1440/1440	Scaffold1	555,518..556957	Aminoglycoside resistance	M13771
*erm(B)*	100	738/738	Scaffold1	557,784..558521	Macrolide resistance	U18931
*cat*	99.85	648/648	Scaffold1	562,873..563520	Phenicol resistance	U35036
*tet(M)*	100	1920/1920	Scaffold2	96,635..98554	Tetracycline resistance	EU182585

## Discussion

### Resistance genes

With sequence similarity alignment, *E. faecalis 2A* (XJ05) was redicted to have resistance to aminoglycoside, macrolide, phenicol and tetracycline. This prediction has been partly confirmed in our studies. Yuan has previously conducted some antibiotic-treatment experiments in 2013 [16]. He found *E. faecalis* could resist gentamicin (aminoglycoside), erythromycin (macrolide), and tetracycline (Tetracycline). Preliminary research from the author’s Laboratory showed that most isolates including *E. faecalis 2A* (XJ05) were resistant to streptomycin, kanamycin and gentamycin(aminoglycoside), tylosin and lincomycin(macrolide), oxytetracycline (tetracycline), phenicol(amphenicols), the drug resistant phenotype of *E. faecalis 2A* (XJ05) coincided with the drug resistant gene it carried [11]. In addition, the strain was also resistant to ampicillin(β-lactam) and sulfadiazine (Sulfonamides), however, no corresponding drug resistance genes were found. Whether this difference was related to other drug-resistant plasmids carried by *E. faecalis 2A* (XJ05) needs to be further studied.

### Cytolysins

Previous studies have shown that pathogenic *E. faecalis* has more combinations of virulence factor genes than that of in normal intestinal flora *E. faecalis* [17] *E. faecalis* in the digestive tract obtains more virulence-related and drug-resistant genes in a some way (plasmid, transposon, bacteriophage). These special *E. faecalis* break through the intestinal mucosal barrier and enter the blood circulation when the external environment changes and the body’s immunity decreases, and finally reach different organs to cause the corresponding infection, such as endocarditis, urinary tract infection, respiratory infection, etc. How to break through the blood-brain barrier to cause encephalitis has not been reported. At the same time, extensive drug resistance makes treatment more difficult. Therefore, the presence of virulence genes and drug resistance genes is an important factor in *E. faecalis* infection. Reference to other *E. faecalis* primers, 2A (XJ05) also carries many known common virulence factor genes, such as *asa1*、*efaA*、*ace*、*esp* 、*cyl*A et al. Cytolysins coded by the hemolysin gene are a class of bacterial toxins that can dissolve cells. Hemolysin belongs to the cytolysin class. As a pore-forming toxin, hemolysin proteins can specifically bind to a complementary immobilized antigen-sensitive erythrocyte or other cells antibodies, that generate the stimulation of surface antigens, and lead to a variety of cells including red blood cells dissolution.

According to the agar hemolysis test, the Streptococcus genus is classified by its ability to dissolve sheep blood cells. The insoluble phenomenon is called γ-hemolysis, the partially dissolved phenomenon is called α-hemolysis, and complete dissolution is called β-hemolysis. Hemolysin has the most serious β-hemolytic effect on human blood. Hemolysin has been shown to inhibit other pathogenic bacteria too [16].

The cytolysin operon is composed of *cylR1*, *cylR2*, *cylLL*, *cylLS*, *cylM*, *cylB*, *cylA* and *cylI* [18]. Among these components, *cylL* is the structural unit of the lysine operon, while *cylM*, *cylB* and *cylA* are responsible for post-translational modification and secretion. They are all situated in *E. faecalis 2A* (XJ05), and only exist in pathogens *2A* (XJ05) and *V583*. Regulatory genes *cylR1*, *cylR2*, and cylI, which induce immune response were not identified in this analysis. The Virulence Finder database (16 Apr. 2015) does not yet include these 3 genes. Previous virulence gene tests showed that strain 2A also carried post-transcriptional expression and secretion genes *cylB* and *cylA* of hemolysin, so *2A* (XJ05) showed significant β-hemolysis.

*E. faecalis* 2A (XJ05) was used to infect lamb, which could also cause encephalitis in mice. Its main pathological feature were different degrees of brain damage, ranging from purulent brain tissue, large infiltrations of neutrophils, vascular congestion, large gaps in nerve cells, neuronal damage, and necrosis. These changes can explain development and symptoms of lamb encephalitis [4, 5]. Most *E. faecalis* isolates from lambs manifested β-hemolysis, some strains showed a reduced level of α-hemolysis [10]. According to Semedo [19], β-hemolytic ability is a gold standard in identifying whether strains produce cytolysin or not. Cytolysin (hemolysin) may play a vital role in the pathogenesis of lamb encephalitis, its more detailed mechanisms needs to be verified by subsequent experiments.

## Conclusion

Given our genome sequence analysis of *E. faecalis 2A* (XJ05) and *V583,* and the comparative analysis of virulence genes from 6 *E. faecalis* strains, we deduced that the occurrence of lamb encephalitis in winter is related to pathogens, environment. During cold winters, the external environment becomes more inhospitable, coupled with weaker innate resistance of lambs. Carrying a variety of virulence-related new genes such as hemolysin gene may be one of the important reasons for the increased virulence of the *E. faecalis 2A* (XJ05). In addition, the presence of multiple and extensive resistance genes is also a cause of increased pathogenicity of *2A* (XJ05).

## Methods

### Source of the *E. faecalis 2A* (XJ05) strain

*E. faecalis 2A* (XJ05) with a lower LD_50_ and Carring a variety of known virulence-related was selected from more than 60 *E. faecalis* which were originally isolated from the brain of dead lambs in Northern Xinjiang, China for genome research. In addition, *E. faecalis 2A* (XJ05) had a extensive drug resistance phenotype to streptomycin, kanamycin, gentamycin, tylosin, lincomycin, oxytetracycline and phenicol.

### Bacterial genomic DNA preparation

*E. facealis 2A* (XJ05) was cultured with brain-heart infusion medium (BHI, Solarbio) and the bacteria were centrifuged, total genomic DNA from strain was extracted according to the conventional methods [6].

### Genome de novo sequencing

A whole genome shotgun strategy was applied for de novo genome sequencing. The genomic DNA of *E. faecalis* 2A (XJ05) was broken into fragments and a library with insert size 345 bp was constructed. Raw genome data were produced by NGS technology using the HiSeq 2000 system (Illumina, USA) at the BGI in Shenzhen, China. The sequencing length was 100 bp paired-ends. Every base was recorded to correspond with a quality score.

### Genome assembly and annotation

Before assembly, all raw data were fitted for high quality. At least 25 tail part that did not score were deleted. Furthermore, those reads whose length did not reach 80 nt were also removed. Based on the de Bruijn method, all high-quality reads were de novo assembled with Soap de novo software. A draft genome and CDS genes were produced and prospered with Climmer (version 3.02), analyzed by COG.

The tRNA, rRNA of the *E. faecalis 2A*(XJ05) were predicted and found with tRNAscan (version 1.23), rRNAmmer (version 1.2) [20]. The sRNA were found through the predicted tRNA compared with data from Rfaml 0.1 (version 10.1) database [21]. The genes in the assembled genome were identified using Glimmer [22]. The genes sequence were compared with the data from KEGG (version: 59), COG (version: 20090331), SwissProt (version: 201206), NR (version: 20121005), GO (version: 1.419) databases et.al through blastx, the coded proteins with the highest sequence similarity of the genes were obtained, thus the annotated information of the genes’s proteins function were analyzed. Genes functions were classified in detail according to the annotation information of NR, GO annotation information of genes and COG database. Specific databases and software [23].

### Comparative genomic studies

All contigs of *E. faecalis 2A* (XJ05) were constructed using a whole genome comparative analysis to *E. faecalis V583* [11], *E. faecalis 62* [13], *E. faecalis D32* [14], *E. faecalis OG1RF* [12] and *E. faecalis Symbioflor 1* [15] with MUSCLE (http:// www. Ebi. ac. uk/ Tools/ msa/ muscle/). A whole genome phylogenetic tree was constructed. Comparison between the genomes was visualized with BRIG.

### Identification of virulence genes and antibiotic resistance genes

Virulence finder (http://cge.cbs.dtu.dk/services/VirulenceFinder/) was used for identification of virulence factors and toxicity ability. ResFinder (http:// cge. Cbs. dtu. dk/ services/ ResFinder/) was used for predicting antibiotic resistance genes (Kleinheinz 2014).

## Abbreviations

BGI: The Beijing Genomics Institute
CDS: Coding Sequence
COG: Cluster of Orthologous Group
*E. faecalis*: *Enterococcus faecalis*
GO: Gene Ontology
KEGG: Kyotoencyclopedia of Genes and Genomes
NGS: Next-Generation Sequencing
NISS: Hospital Infection Surveillance System
NR: RefSeq non-redundant proteins
ORF: Open Reading Frame
SignalP: Signal peptide
TMHMM: TransMembrane prediction using Hidden Markov Models
